# Air pollution perception in ten countries during the COVID-19 pandemic

**DOI:** 10.1007/s13280-021-01574-2

**Published:** 2021-06-21

**Authors:** Baowen Lou, Diego Maria Barbieri, Marco Passavanti, Cang Hui, Akshay Gupta, Inge Hoff, Daniela Antunes Lessa, Gaurav Sikka, Kevin Chang, Kevin Fang, Louisa Lam, Brij Maharaj, Navid Ghasemi, Yaning Qiao, Solomon Adomako, Ali Foroutan Mirhosseini, Bhaven Naik, Arunabha Banerjee, Fusong Wang, Andrew Tucker, Zhuangzhuang Liu, Kasun Wijayaratna, Sahra Naseri, Lei Yu, Hao Chen, Benan Shu, Shubham Goswami, Prince Peprah, Amir Hessami, Montasir Abbas, Nithin Agarwal

**Affiliations:** 1 School of Highway, Chang’an University, Nan Er Huan Road (Mid-section), Xi’an, 710064 Shaanxi China; 2 Department of Civil and Environmental Engineering, Norwegian University of Science and Technology, Høgskoleringen 7A, 7491 Trondheim, Trøndelag Norway; 3 Italian Society of Cognitive Behavioural Therapy (CBT-Italy), Mannelli St. 139, 50132 Firenze, Toscana Italy; 4 Centre for Invasion Biology, Department of Mathematical Sciences, Stellenbosch University, Matieland, 7602 South Africa; 5 Biodiversity Informatics Unit, African Institute for Mathematical Sciences, Cape Town, 7945 South Africa; 6 Department of Civil Engineering, Transportation Engineering Group, Indian Institute of Technology Roorkee, 321-A&B, Roorkee, Uttarakhand 247667 India; 7 Department of Civil Engineering, Federal University of Ouro Preto, Rua Nove, Bauxita, Ouro Preto, Minas Gerais 35400-000 Brazil; 8 Department of Geography, Lalit Narayan Mithila University, Darbhanga, Bihar 846004 India; 9 Department of Civil and Environmental Engineering, University of Idaho, 875 Perimeter Drive, Mailstop 1022, Moscow, ID 83844 USA; 10 Department of Geography, Sonoma State University, Environment, and Planning, 1801 East Cotati Avenue, Rohnert Park, CA 94928 USA; 11 School of Health, Federation University Australia, 72-100 Clyde Rd, Berwick, VIC 3806 Australia; 12 Department of Geography, University of KwaZulu-Natal, Howard College City, Durban, 4000 KwaZulu South Africa; 13 Department of Civil Chemical Environmental and Materials Engineering, University of Bologna, Viale del Risorgimento, 2, 40136 Bologna, Emilia-Romagna Italy; 14 School of Mechanics and Civil Engineering, China University of Mining and Technology, Daxue Road 1, Xuzhou, 22116 Jiangsu China; 15 Department of Engineering and Science, University of Agder, Jon Lilletuns vei 9, 4879 Grimstad, Agder Norway; 16 Department of Civil Engineering/Russ College of Engineering & Technology, Ohio University, 28 W. Green Drive, Athens, OH 45701 USA; 17 Department of Civil Engineering, Indian Institute of Technology Guwahati, Guwahati, Assam 781039 India; 18 State Key Laboratory of Silicate Materials for Architectures, Wuhan University of Technology, Luoshi road 122, Wuhan, 430070 Hubei China; 19 Connecticut Transportation Safety Research Center, University of Connecticut, 270 Middle Turnpike, Unit 5202 Longley Building, Storrs, CT 06269 USA; 20 School of Civil and Environmental Engineering, University of Technology Sydney, 81, Broadway, Ultimo, NSW 2007 Australia; 21 School of Medicine, Bam University of Medical Sciences, Bam, 76615-336 Kerman, Iran; 22 School of Civil Engineering, Sun Yat-Sen University, Xingang Xi Road 135, Guangzhou, 510275 Guangdong China; 23 Foshan Transportation Science and Technology Co. Ltd., Kuiqi Second Road 18, Foshan, 528000 Guangdong China; 24 Department of Civil Engineering, Indian Institute of Science Bangalore, C V Raman Avenue, Bangalore, Karnataka 560012 India; 25 Department of Social Policy Research Centre, University of New South Wales, John Goodsell Building, Kensington, Sydney, NSW 2052 Australia; 26 Department of Civil and Architectural Engineering, Texas A&M University – , Kingsville, 917 W. Ave B, Kingsville, TX 78363 USA; 27 Department of Civil and Environmental Engineering, Virginia Tech, 301-D3 Patton Hall, Blacksburg, VA 24061 USA; 28 Department of Civil & Coastal Engineering, University of Florida, 2100 NE Waldo Rd., Sta 106, Gainesville, FL 32609 USA

**Keywords:** Air quality, COVID-19 pandemic, Environmental pollution, Pollution perception, Psychometric perception

## Abstract

As largely documented in the literature, the stark restrictions enforced worldwide in 2020 to curb the COVID-19 pandemic also curtailed the production of air pollutants to some extent. This study investigates the perception of the air pollution as assessed by individuals located in ten countries: Australia, Brazil, China, Ghana, India, Iran, Italy, Norway, South Africa and the USA. The perceptions towards air quality were evaluated by employing an online survey administered in May 2020. Participants (*N* = 9394) in the ten countries expressed their opinions according to a Likert-scale response. A reduction in pollutant concentration was clearly perceived, albeit to a different extent, by all populations. The survey participants located in India and Italy perceived the largest drop in the air pollution concentration; conversely, the smallest variation was perceived among Chinese and Norwegian respondents. Among all the demographic indicators considered, only gender proved to be statistically significant.

**Supplementary Information:**

The online version contains supplementary material available at 10.1007/s13280-021-01574-2.

## Introduction

### Background

Air pollution is a global environmental issue, which has been steadily increasing during the last decades due to urban sprawl and anthropogenic activities (Yang et al. 2018; Li et al. 2019a) causing severe health diseases (Lelieveld et al. 2015; Cohen et al. 2017; Burnett et al. 2018) and reducing people’s Subjective Well-Being (SWB) to a significant degree (Li et al. 2018). On average, approximately 4 million deaths per year can be linked to poor air quality and pollutants (i.e. particulate matter PM, which is usually referred to according to an aerodynamic diameter of less than 2.5 μm PM_2.5_ or 10 μm PM_10_, ozone O_3_, nitrogen oxides NO_x_, carbon monoxide CO and sulphur dioxide SO_2_), especially in major developing countries (WHO 2016).

Facilitated by globalisation and our hypermobile society (Acter et al. 2020; SanJuan-Reyaes et al. 2020; Sarkar et al. 2020), the COVID-19 pandemic has become another grave issue for humanity as a whole, forcing radical changes in many social, economic and hygienic behaviours (WHO 2020a, b; Passavanti et al. 2021; Wu 2021). In order to curb the spread of the COVID-19 virus, a significant amount of the global population has been requested to comply with restrictions to economic and mobility activities (De Vos 2020; Wilder-Smith and Freedman 2020; Barbieri et al. 2021). Although essential industries have been operating continuously (Wang et al. [Bibr CR139][Bibr CR141]), the massive decline in the global pattern of energy demand (i.e. crude oil and coal) and the general slowdown of anthropogenic activities have involuntarily imposed a unique scenario curtailing detrimental emissions released into the troposphere (Berman and Ebisu 2020; Kumari and Toshniwal 2020; Shi and Brasseur 2020) offering “the nature a healing time” (Lokhandwala and Gautam 2020).

Unlike other sudden large-scale drops in air pollution recorded previously in relation to particular events (Li et al. 2019b), such as the 1996 Atlanta Olympics (Friedman et al. 2001), the 2008 Beijing Olympics (Huang et al. 2012) and the 2014 Asia–Pacific Economic Cooperation meeting (Wang et al. 2017), the geographical extent of the effects exerted by the COVID-19 pandemic has been global. In this regard, there are numerous studies, which performed robust chemical and meteorological analyses, documenting the reduction in air pollution during the pandemic for a variety of noxious particles and gases, i.e. particulate matter PM_2.5_ (Chauhan and Singh 2020; Rodríguez-Urrego and Rodríguez-Urrego 2020), nitrogen dioxide NO_2_ (ESA 2020; NASA [Bibr CR97][Bibr CR98][Bibr CR99]; Venter et al. 2020; Liu et al. 2021), carbon monoxide CO (Dantas et al. 2020; Barua and Nath 2021) and carbon dioxide CO_2_ (Le Quéré et al. 2020; Andreoni 2021) and a moderate decrease in Aerosol Optical Depth (Lal et al. 2020; Muhammad et al. 2020).

### Scope of the study

Unique to the number of existing studies focussing primarily on measured environmental implications of the pandemic-related restrictions (Shakil et al. 2020), this research addresses a topic that has been often neglected, namely assessing the human perceptions towards air quality and its change. In particular, we investigate the perceptions related to air pollution experienced by individuals located in ten countries: Australia, Brazil, China, Ghana, India, Iran, Italy, Norway, South Africa and the USA (hereafter, also referred to by their acronyms AU, BR, CH, GH, IN, IR, IT, NO, ZA and USA, respectively).

Following previous psychometric investigations dealing with perceptions of air quality (Nikolopoulou et al. 2011; Pu et al. 2019; Reames and Bravo 2019) and psychological impacts on people’s Subjective Well-Being (Li et al. 2018) by means of opinion surveys, this study captures the perceptions towards atmospheric quality related to before and during the enactment of the pandemic-related restrictions (Barbieri et al. 2020b).

Previous psychometric researches highlighted that several factors, ranging from social, personal, political to cultural dimensions, can affect the air quality perception. At the same time, the information regarding environmental pollution conveyed to the general public may not always result transparent because of issues related to information source (i.e. biased coverage) or information receiver (i.e. difficulty in understanding highly technical content). Compounding this, it is unclear the extent according to which information and awareness about air pollution can effect actual behavioural changes (Oltra and Sala 2014). We employed an online survey administered in the abovementioned ten countries in May 2020. This approach allows for a comparison, at a cross-country scale, of how air quality has been perceived by residents with various demographics facing different levels of air pollution before the COVID-19 pandemic.

The detrimental health effects related to the exposure to particulate matter PM (Puett et al. 2009; Hoek et al. 2013; Hamra et al. 2014; Stafoggia et al. 2014) and ozone O_3_ ( Ito et al. 2005; Nuvolone et al. 2018; Feng et al. 2019; Siciliano et al. 2020b) are largely believed to be the most hazardous form of air pollution (WHO 2006). Therefore, considering the relevance of PM_2.5_, PM_10_, O_3_ for both physical and psychological well-being (Rotko et al. 2002; Li et al. 2018), we investigate the level of variations in the pollutants concentration that are likely to trigger perceptual changes.

## Materials and methods

### Research on air pollution perception

The earliest studies encompassing people’s perceptions on air pollution were performed in the 1960s and were largely quantitative and evaluated the extent of public awareness on diverse environmental issues (Smith et al. 1964; Schusky 1966; de Groot 1967; Crowe 1968). As the perception of air pollution represents a multifaceted topic, starting from the 1990s a new body of research started to adopt qualitative methods in two areas: (i) understanding the demographic, social and cultural determinants related to the interpretation and the perception of air pollution (Bickerstaff and Walker 1999; Bush et al. [Bibr CR28][Bibr CR29]) and (ii) enhancing communication in a reliable and trustworthy fashion to stimulate public behavioural changes (Beaumont et al. 1999; Howel et al. 2003).

The general improvement in air pollution during the COVID-19 pandemic has received wide coverage in the news and other journalistic platforms, thus inspiring growing discussions among the general public on social media and websites (Brimblecombe and Lai 2020; Casado-Aranda et al. 2020; Alshaabi et al. 2021). This phenomenon is in line with previous large-scale events which stimulated the rise of environmentalism in different locations across the globe (Brimblecombe and Zong 2019). The psychological effects of air pollution (i.e. anxiety, depression, distress, nuisance, impairments in concentration), generally less investigated than the physical effects, are a crucial factor to successful environmental policies for addressing pollution abatement (Deguen et al. 2012); in addition, few studies have involved developing countries (Saksena 2011). In general, females of all age classes living in urban areas and with higher education represent the part of the population most concerned about environmental issues (Oltra and Sala 2014). Nevertheless, the lack of awareness about the sources of pollution and its consequences is present across various socio-economic groups and often entails underestimation of objective reality (Oltra and Sala 2014; Maione et al. 2021).

### Survey investigation

An online survey was developed and administered to evaluate the public perceptions of the quality of air before and during the COVID-19 restrictions enforced in each of the ten countries (Australia, Brazil, China, Ghana, India, Iran, Italy, Norway, South Africa and the USA) allowing for a cross-sectional study. Respondents expressed their opinions according to a 7-point Likert-scale question with “1 = extremely low/absent air pollution” and “7 = extremely high air pollution”. The questionnaire also collected information regarding gender, age and education of the participants (Barbieri et al. 2020b).

The web-based survey in this study was created with Google Forms and WenJuanXing (translated into Chinese, English, Italian, Norwegian, Persian, Portuguese) and distributed between the 11th and the 31st of May 2020 by means of professional and social networks (including but not limited to email lists, Facebook, LinkedIn, Twitter, Instagram, Skype, WhatsApp, WeChat, Weibo, QQ and Douban) using purposive sampling technique (De Beuckelaer and Lievens 2009; Stockemer 2019); more respondents were obtained via snowball sampling through the forwarding and sharing the survey by initial recipients. The linguistic validity across the ten countries was pursued following a translation-back-translation approach (Brislin 1976): after translating the survey into local languages, the survey was back translated. The research team carefully addressed and resolved all the discrepancies to ensure full linguistic equivalence. The survey was approved by two major institutional review boards (Norwegian Centre for Research Data and Ohio University Office of Research Compliance). Informed consent was obtained from all respondents consistent with the Declaration of Helsinki.

The COVID-19 response stringency index (Oxford University 2020) shows that most countries worldwide had implemented their most restrictive policies by the 11th of May with the largest part of the global population facing some form of lockdown (Barbieri et al. 2020a; Sovacool et al. 2020). As several studies in cognitive psychology on human memory have indicated possible distortions and difficulty of recall from forgotten or telescoped events (Coughlin 1990; Solga 2001; Barsky 2002; Jaspers et al. 2009), responses to retrospective questions are considered reliable only for a relatively short period, generally ranging from some days to about a year (Smith 1984; Hipp et al. 2020). Therefore, by undertaking the survey in May 2020, it is assumed that all the survey participants were able to properly compare the air pollution “before” (retrospective question) and “during” (current question) the pandemic thanks to the short amount of time, ranging from some weeks to very few months, between the enforcement of the restrictions and the administration of the survey.

### Performed analyses

The individual perceptions about the air pollution expressed according to the 7-point Likert scale were analysed and compared at a cross-country level. Furthermore, statistical analyses probed any possible correlations existing between the survey responses and the three demographic indicators considered (gender, age and education). The calculations were performed with the software package IBM SPSS Statistics version 27. The regression method employed was the Negative Binomial Model (NBM): NBM is a Generalised Linear Model and was selected as the hypotheses necessary to achieve simpler analyses (i.e. linear regression or ANOVA) were not fulfilled (i.e. normality of the residuals) (Ajide et al. 2020). Gender and education were regarded as categorical independent variables and age was treated as a continuous independent variable.

To test the extent to which the changes in air pollution related to PM_2.5_, PM_10_ and O_3_ are likely to trigger changes in air pollution perception, we also collected data for these air pollutants for two temporal frames, namely 01.01.2019–31.05.2019 and 01.01.2020–31.05.2020. As individual retrospective perceptions are most likely valid for a relatively short period as explained above, the pollutant concentrations were evaluated only for the 1-year time span. Consequently, the well-known interannual differences in atmospheric circulation, meteorology and emission sources were not analysed as part of this study.

The intensity of air pollution as a whole is expressed by an indicator called Actual Air Pollution Quantity (AAPQ). AAPQ is a weighted combination of the three considered air pollutants PM_2.5_, PM_10_ and O_3_ and is assessed by means of Principal Component Analysis (PCA), which is an orthogonal transformation employed to reduce the dimensionality of complex datasets to a lower dimension (Hotelling 1933).

The concentrations of PM_2.5_, PM_10_, O_3_ were retrieved from national Environmental Protection Agencies (EPAs) and national monitoring centres for each of the ten countries involved in this study, namely Australia (EPA South Australia 2020; EPA Victoria 2020; NSW Office of Environment and Heritage 2020), Brazil (CETESB – Companhia Ambiental do Estado de São Paulo 2020; CETREL 2020), China (China National Environment Monitoring Centre 2020), Ghana (AirNow Department of State 2020), India (CPCB Central Pollution Control Board 2020), Iran (Department of Environment Iran 2020), Italy (ARPA Emilia-Romagna 2020; ARPA Lombardia 2020; ARPA Piemonte 2020), Norway (Luftkvalitet i Norge 2020), South Africa (SAAQIS South African Air Quality Information System 2020) and the USA (United States Environmental Protection Agency 2020). All measurements were derived from 1 043 ground-based stations located in the regions/states/provinces/counties matching the geographical locations of the survey respondents.

## Results and discussion

### Reach of the survey

**Fig. 1:**
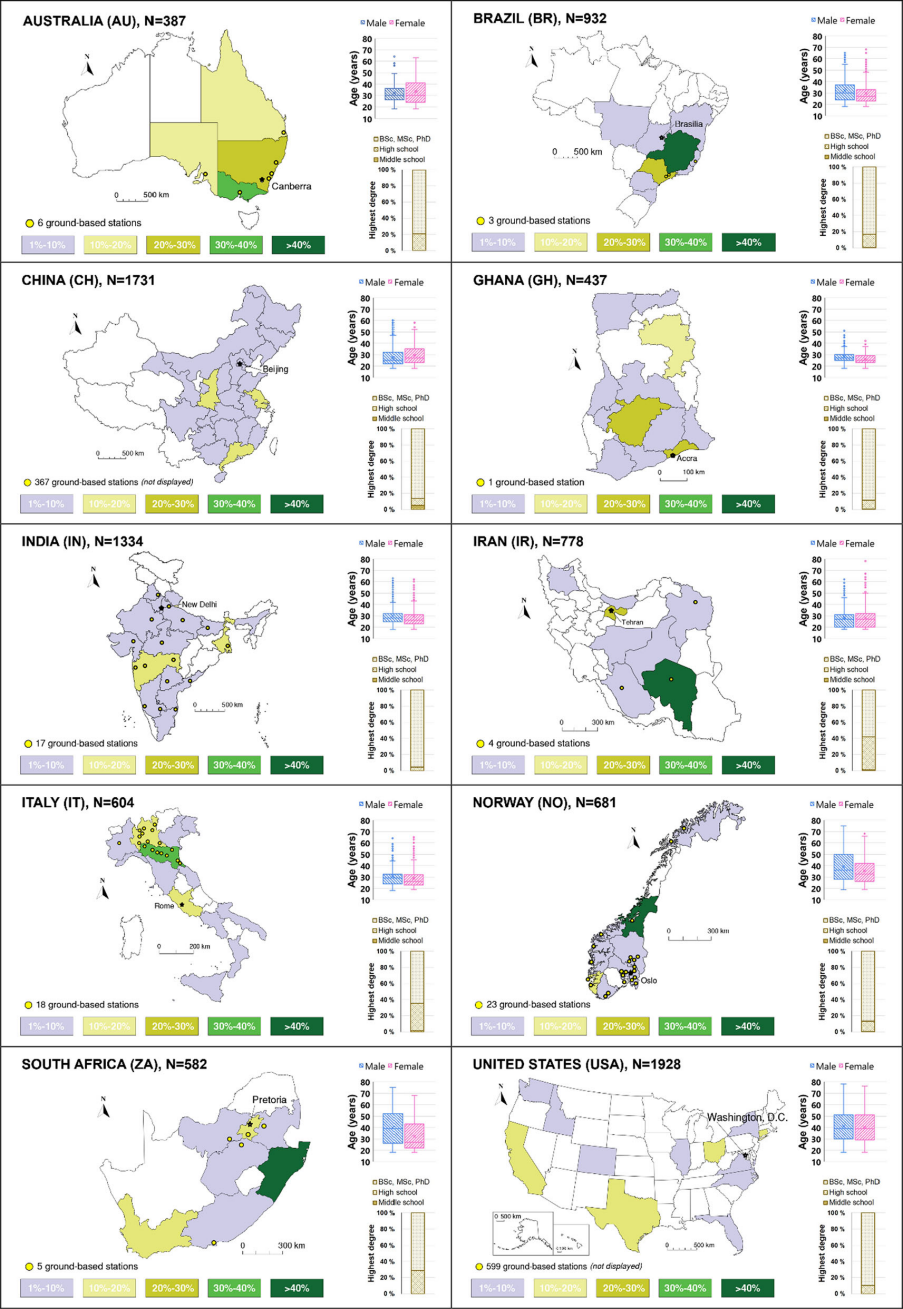
Sample size, geographical distribution of respondents for each country (percent), age, gender and education split. Locations of ground-based monitoring stations (not displayed for China and the USA)

The geographical distribution and the demographic information of the survey respondents are depicted in Fig. 1. The online survey included a balanced representation of gender (male 50.9% and female 48.9%) with a total of 9 394 participants. Respondents tended to be younger and middle-aged adults (*M* = 32.6, SD = 11.6) and were also largely comprised of those with higher levels of education (81.3% held at least a bachelor’s degree). Thus, the results here likely reflected changes in perceptions of middle class individuals with probable better awareness of issues pertinent to air pollution (Bickerstaff and Walker 2001), particularly in less wealthy countries where internet access to the online-administered survey is less ubiquitous. The survey sample, albeit substantial, was skewed from the overall population demographic composition and, therefore, should only be considered as tentatively indicative of the actual perception of the general public. The survey dataset formed is publicly available (Barbieri et al. 2020b).

Figure 1 also reports on the locations of all the ground-based monitoring stations adopted to retrieve data on the concentration of PM_2.5_, PM_10_ and O_3_. The positions of the monitoring stations for China and the USA are not shown on the map due to their large numbers (367 and 599, respectively), which would cover the entire areas shaded in the figure. Only one ground-based station was available in Ghana to monitor the amount of particulate matter in 2020.

### Perceived pollution

**Fig. 2:**
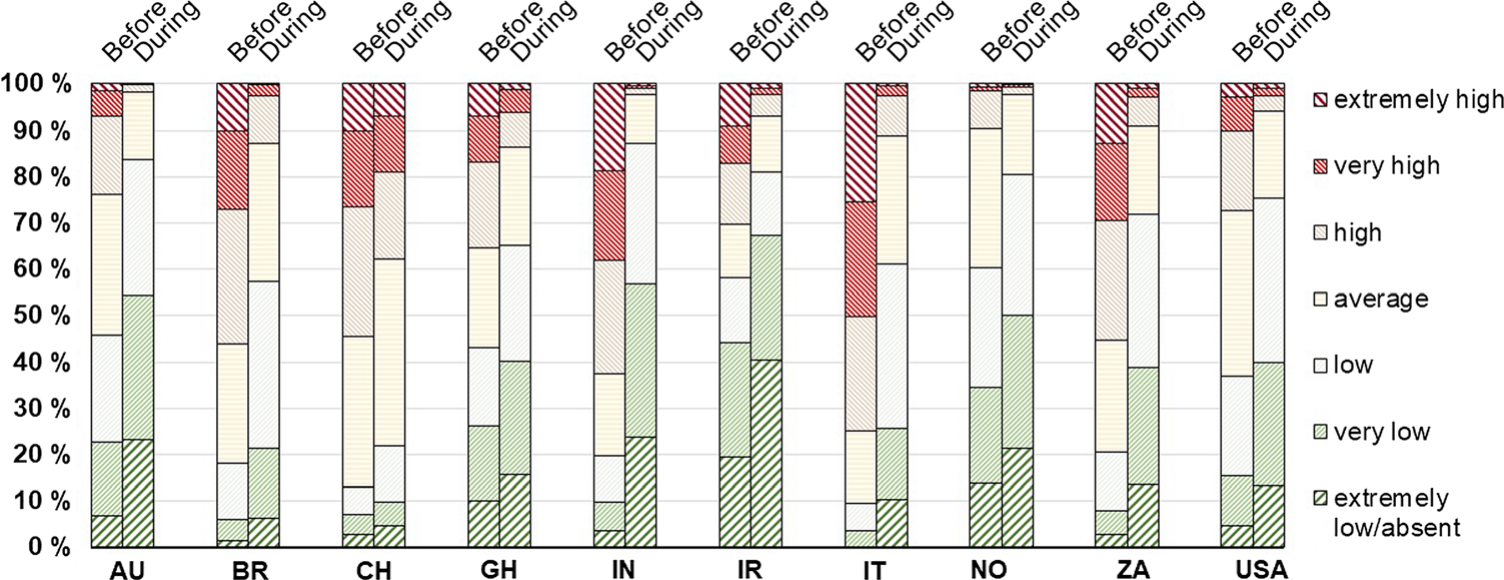
Perceived Air Pollution Quantity (PAPQ) before and during the pandemic-related restrictive measures by the survey respondents

Considering the responses associated to the Likert scale varying from “1 = extremely low/absent” to “7 = extremely high”, a general improvement in atmospheric quality was clearly perceived in all ten investigated countries (*M *= 4.08, SD = 1.61 before restrictions, *M *= 2.84, SD = 1.28 during restrictions), albeit to different extents as reported in Fig. 2. The perceptions are in line with other studies shedding light on the quantitative improvement in air quality, such as in Brazil (Dantas et al. 2020; Nakada and Urban 2020; Siciliano et al. [Bibr CR123][Bibr CR123]), China (Chen et al. [Bibr CR35][Bibr CR36]; Le et al. 2020; Li et al. 2020; Sicard et al. 2020; Wang and Su 2020), India (Lokhandwala and Gautam 2020; Mahato et al. 2020; Sharma et al. 2020; Singh et al. 2020; Srivastava et al. 2020; Yadav et al. 2020; Mishra et al. 2021), Iran (Ahmadi et al. 2020), Italy (Collivignarelli et al. 2020; Rugani and Caro 2020; Sicard et al. 2020) and the USA (Bashir et al. 2020; Berman and Ebisu 2020; Chen et al. 2020).

Compared to before the restrictions, the number of individuals describing the air pollution as “low”, “very low” or “extremely low/absent” increased more than 3 times in Brazil, India, Italy and South Africa. Moreover, the amount of respondents reporting “extremely low/absent” changed from 3.5% to 23.9% (approximately 7 times) in India and from 0.2% to 10.3% (approximately 60 times) in Italy, respectively. Accordingly, the number of individuals depicting the level of atmospheric pollution as “high”, “very high” or “extremely high” was significantly reduced, even in those countries where air pollution was perceived to be low before the pandemic (such as Australia and Norway). People’s perceptions on air pollution reduction differ significantly between countries (Chi square test; *p* < .001).

**Fig. 3:**
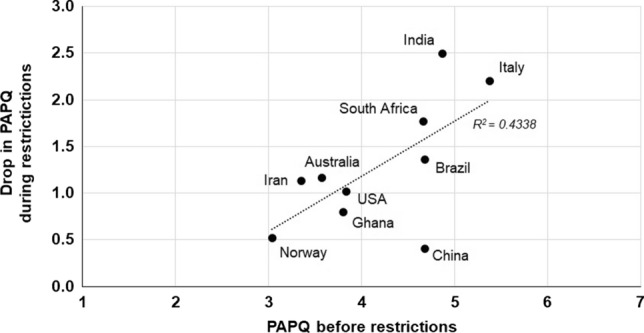
Perceived Air Pollution Quantity (PAPQ) before the restrictions and drop in PAPQ during the restrictions by the survey respondents (Likert-type scoring system varying from “1 = extremely low” to “7 = extremely high”)

The mean responses were further considered; as reported in Fig. 3, the data points corresponding to those countries where respondents perceived a larger amount of pollution before the pandemic accounted for a larger drop in the Perceived Air Pollution Quantity (PAPQ) during the enactment of the restrictions. The fact that individuals from countries of greater atmospheric pollution perceived a much greater air quality improvement can be associated to the fact that human attitudes and decisions may not always be based on factuality, but on baseline conditions according to the theory of irrational perception and decision making (Tversky and Kahneman 1981; Kahneman and Tversky 1996; Bickerstaff 2004). Alternatively, this could also be due to the fact that individual experience can dictate current perceptions, known as the “hot-stove effect” (Graves 2003; Denrell and March 2001). The largest drop in PAPQ during the restriction was from respondents in India and Italy; on the other hand, Norwegian and Chinese survey participants perceived the smallest drop in PAPQ. In Fig. 3 the data point representing Chinese respondents is the farthest to the regression line (*R*^2^ = 0.4338). This represents an unexpected outcome considering the high pollution level of the country (Tong et al. 2014; Guo et al. 2016) and could reflect a legacy effect from the combination of long-term exposure to poor quality air and the lack of awareness (Huang and Yang 2020).

### Role of gender, age, education

Considering the results of the statistical analyses displayed in Table 1, gender was the only significant predictor (*p* < .05). As depicted in Figure S1, females generally perceived more air pollution. As for the other two variables education and age, no significant correlations were found. No collinearity issues between the three independent variables (gender, education and age) were detected while performing the analyses. Based on the existing literature, there appears to be a lack of univocal support regarding the significant demographic predictors of air pollution perception. The importance of gender as emerged in this study is in agreement with other investigations performed in different places of the globe also focussing on the same topic (Rotko et al. 2002; De Feo et al. 2013; Liao et al. 2015; Chakraborty et al. 2017; Cisneros et al. 2017). Contrastingly, previous studies demonstrated a significant correlation of pollution perception to education level (Klæboe et al. 2000; Badland and Duncan 2009) or age (Lercher et al. 1995; Liu et al. 2016) or all of the three social indicators (Lai and Tao 2003), while some investigations found no gender bias in perceptions of environmental concerns (Howel et al. 2003; Kim et al. 2012; Omanga et al. 2014; Becken et al. 2017).

**Table 1:** Likelihood ratio Chi Square, deviance/df ratio, parameters estimates, standard deviation, and statistical significance (B ± S.E.^x^) for the responses on perceived pollution before and after the enactment of the pandemic-related restrictions

	Before restrictions	During restrictions
Likelihood ratio Chi Square	12.704	15.669
Deviance/df ratio	0.150	0.162
Parameters estimates		
Male | Female	− 0.065 ± 0.023^**^	− 0.051 ± 0.024^*^
Education 1 | 6	0.118 ± 0.298^ns^	0.284 ± 0.304^ns^
Education 2 | 6	− 0.019 ± 0.118^ns^	0.212 ± 0.12^ns^
Education 3 | 6	− 0.010 ± 0.049^ns^	0.002 ± 0.051^ns^
Education 4 | 6	0.032 ± 0.041^ns^	0.068 ± 0.043^ns^
Education 5 | 6	0.054 ± 0.042^ns^	0.029 ± 0.044^ns^
Age	− 0.001 ± 0.001^ns^	− 0.002 ± 0.001^ns^

*ns* non-significant, *“Education 1”* Primary school, *“Education 2”* Middle school, *“Education 3”* High school, *“Education 4”* BSc, *“Education 5”* MSc, *“Education 6”* PhD

*p < 0.05, **p < 0.01

### Indicative comparison of actual and perceived pollution

The levels of air pollutants PM_2.5_, PM_10_ and O_3_ were extrapolated; the data retrieved were available on a 1-hour, 8-hour or 24-hour basis depending on each monitoring station. Average pollution levels in 2019 and 2020 are summarised in Table 2. From year to year, there were substantial reductions in particulate matter in eight countries. Conversely, Australia saw increments in both PM_2.5_ and PM_10_, most likely due to extremely high concentrations in January 2020 concurrent with the severe bushfire season (Jalaludin et al. 2020). In general, PM_2.5_ had a deeper decline than PM_10_: the average decreases, assessed from all the ten countries, were -21.03 μg/m^3^ and -4.98 μg/m^3^, respectively. The most significant drops were registered in India for PM_2.5_ (-94.79 μg/m^3^) and in China for PM_10_ (-14.93 μg/m^3^). For ozone, concentrations generally increased in 2020 with respect to 2019. Considering mean values, the largest hikes were registered in the USA (+59.03 μg/m^3^) and Norway (+44.38 μg/m^3^). Increases in ozone concentrations are not necessarily inconsistent with overall better air quality and the reduction of other pollutants (Sillman and He 2002; Li et al. [Bibr CR82][Bibr CR83][Bibr CR84]; Mahato et al. 2020; Siciliano et al. 2020b).

**Table 2:**
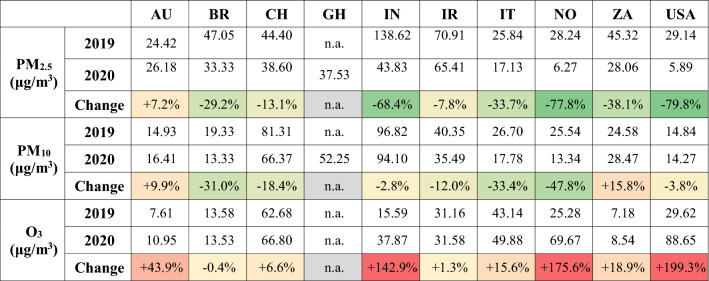
Average values of particulate matter PM_2.5_, PM_10_ and ozone O_3_ evaluated between the 1st of January and the 31st of May in 2019 and in 2020

*n.a.* not available

**Fig. 4:**
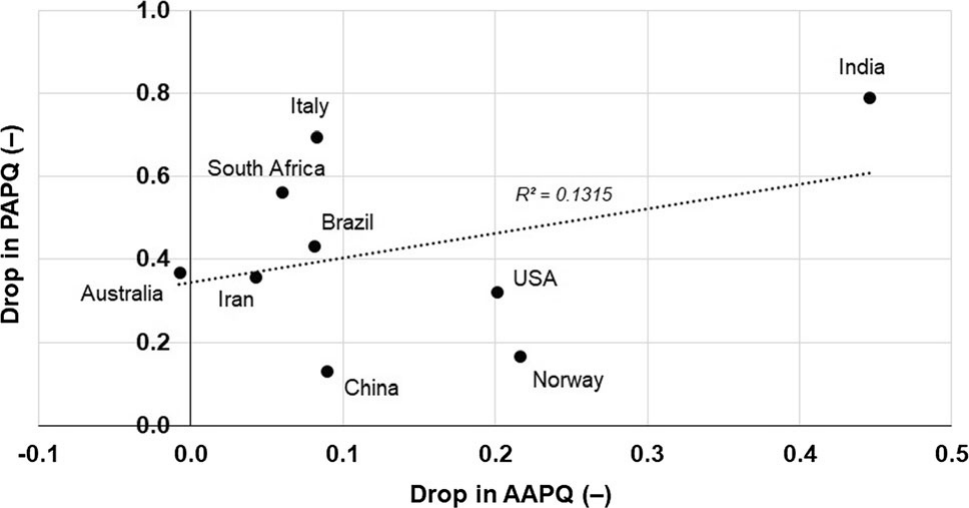
Comparison between drop in Perceived Air Pollution Quantity (PAPQ) and drop in Actual Air Pollution Quantity (AAPQ)

Considering the drop in PAPQ versus the registered drop in AAPQ (Fig. 4), a positive and weak correlation is found (R^2^ = 0.1315). In addition, the relationships between the variation in concentration of each pollutant and PAPQ are reported in Figure S2 as separate entities to match the initial source categories.

It must be noted that there are certain limitations related to the calculations of PM_2.5_, PM_10_ and O_3_ performed in this study. Pollutant concentrations may fluctuate according to the change in atmospheric circulation due to seasonal disparity between southern and northern hemispheres, while locations with different irradiation and rainfall patterns may be related to specific wet scavenging processes of air pollutants (Elperin et al. 2011; Berman and Ebisu 2020). Moreover, as it was not possible to assess the precise distance between survey respondents and ground-based monitoring stations, no inverse distance weighting could be applied to adjust the exposure. Therefore, the findings connecting the variation in the pollutants concentration and the individual perceptions should be considered indicative only. There was not enough data available for Ghana to be included in the discussion of results.

## Conclusions

Activities from human settlements are responsible for significant amounts of pollution externalities, which in turn lead to physical and psychologic detrimental effects on human well-being. This study focussed on the perception of the air pollution in ten countries (Australia, Brazil, China, Ghana, India, Iran, Italy, Norway, South Africa and the USA) in conjunction with the reduction in the hazardous emissions released into the troposphere during the enforcement of the COVID-19 pandemic restrictions. An online survey administered in May 2020 collected information about the level of air pollution perceived by individuals (*N* = 9 394) before and during the COVID-19 mitigation measures. The following conclusions can be drawn:

1. Albeit at different extents, all survey respondents expressed a significant improvement in the air quality and such positive public perception should be considered a motivation for long-term systemic change for mitigating air pollution worldwide. The most striking decreases in poor air quality were perceived in India and Italy. Conversely, the smallest variations were perceived among Chinese and Norwegian respondents (see reason below).
2. People from different countries did experience an improvement in air quality in conjunction with the implementation of the pandemic-related mitigation measures. However, considering the central role of publicity around air pollution as a crucial factor for stimulating public awareness, individuals are likely to underestimate the improvement in air quality nor to identify the level of air pollution in an unbiased fashion.
3. Among the demographic indicators considered, the air quality perceptions of the surveyed population strongly hinged upon one factor: gender. Compared to male respondents, female respondents perceived a higher amount of air pollution, both before and during the pandemic-related restrictions. Neither education nor age were found to be significant sociodemographic indicators for air pollution perception.
4. Based on the indicative comparison performed between the levels of actual and perceived atmospheric pollution, a positive and weak relationship was found. Therefore, being the pollution experienced as a personal combination of olfactory and visual impacts, individuals are not amenable to perceive air quality objectively.

The remarkable reduction in air pollution during the COVID-19 pandemic may just be temporary and may revert to previous trends if both the citizenry and policymakers do not realise the “pervasive, omnipresent and interdependent” lessons learned (Bergman 2020; McNeely 2021). The cross-sectional nature of this study prevented the opportunity to disentangle whether the respondents’ perceptions were biased by local and global media coverage or specific neighbourhood characteristics (Sax et al. 2003; Earl et al. 2004; Oltra and Sala 2014). The findings indicate that policies and strategies for air quality enhancement must be planned strategically with the realisation that public acceptance may not be straightforward and can be gender dependent. Further environmental parameters, which may affect the overall comfort of the individuals, can be taken into consideration in future research to delve into the behavioural impacts and the psychological consequences of air pollution.

## Supplementary Information

Below is the link to the electronic supplementary material.

Supplementary file1 (PDF 524 kb)
