# Supplementary material for: Air pollution perception in ten countries during the COVID-19 pandemic
Source: Ambio. 2021 Jun 21;51(3):531–45. doi: 10.1007/s13280-021-01574-2 (PMC8216327; doi:10.1007/s13280-021-01574-2)
Supplement: Supplementary file 1 — Supplementary file1 (PDF 524 kb) [file 13280_2021_1574_MOESM1_ESM.pdf]

**Ambio**

Electronic Supplementary Material
*This supplementary material has not been peer reviewed.*

Title: Air pollution perception in ten countries during the COVID-19 pandemic

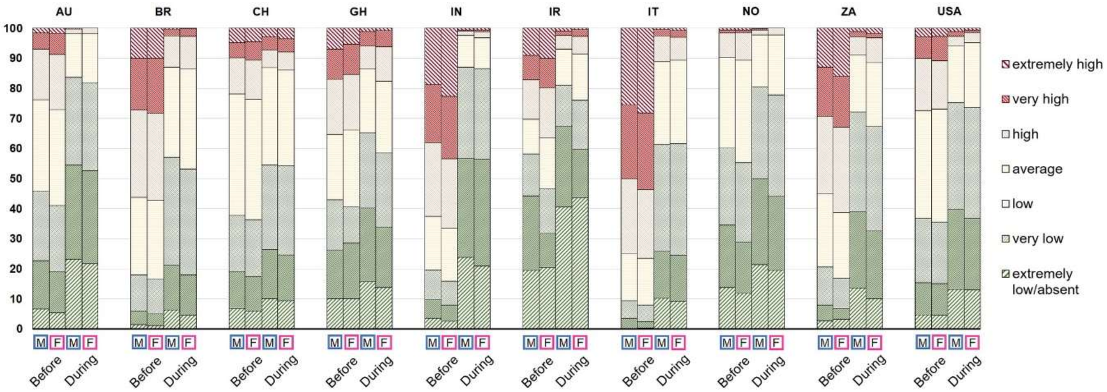


Fig. S1. Perceived Air Pollution Quantity (PAPQ) before and during the pandemic-related restrictive measures by the survey respondents subdivided by gender: male (M) and female (F).

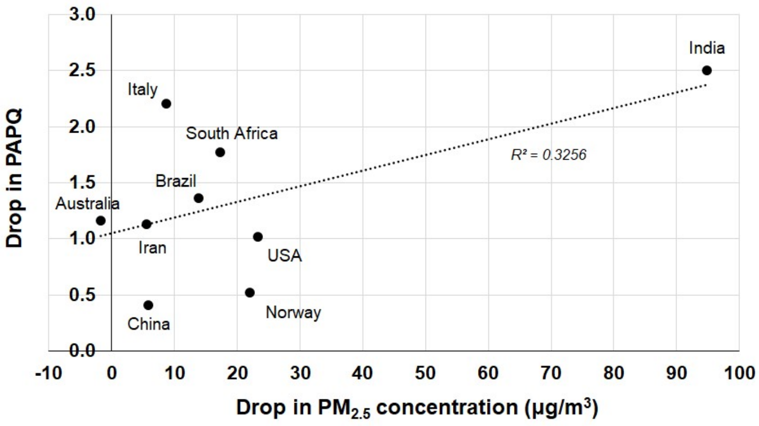


(a)

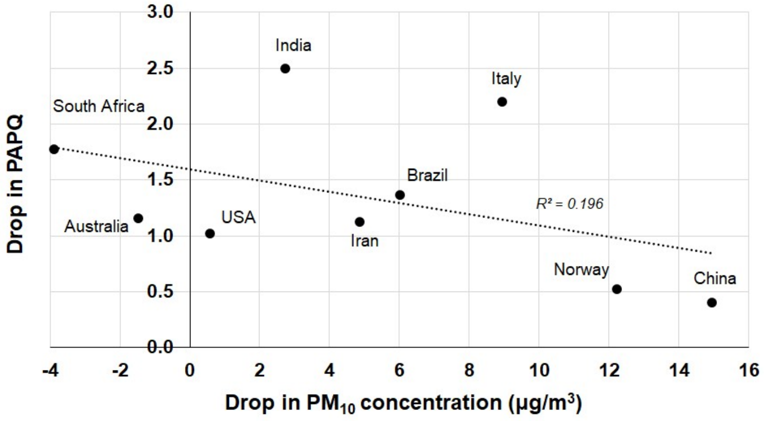


(b)

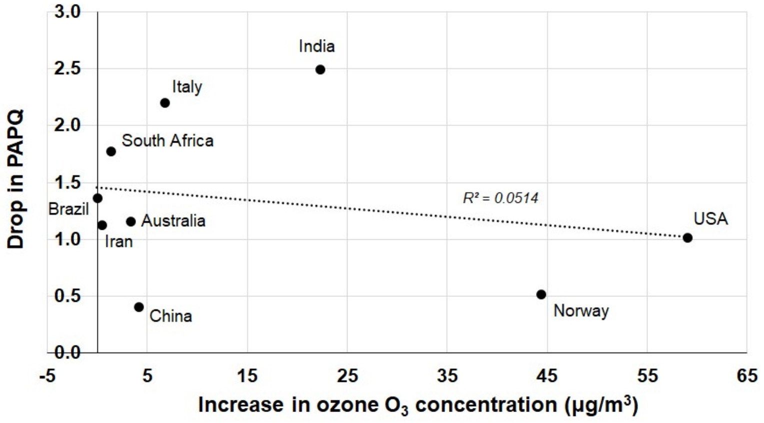


(c)

Fig. S2. Drop in Perceived Air Pollution Quantity (PAPQ) and variation in the concentration of particulate matter $PM_{2.5}$ (a), $PM_{10}$ (b), ozone $O_3$ (c) retrieved from ground-based stations.
